# Environmentally Safe Photodynamic Control of *Aedes aegypti* Using Sunlight-Activated Synthetic Curcumin: Photodegradation, Aquatic Ecotoxicity, and Field Trial

**DOI:** 10.3390/molecules27175699

**Published:** 2022-09-04

**Authors:** Alessandra R. Lima, Cicera M. Silva, Lucas M. da Silva, Amilcar Machulek, Antônio P. De Souza, Kleber T. de Oliveira, Larissa M. Souza, Natalia M. Inada, Vanderlei S. Bagnato, Samuel L. Oliveira, Anderson R. L. Caires

**Affiliations:** 1 Institute of Physics, Federal University of Mato Grosso do Sul, Campo Grande 79070-900, Brazil; 2 São Carlos Institute of Physics, University of São Paulo, São Carlos 13566-590, Brazil; 3 Institute of Chemistry, Federal University of Mato Grosso do Sul, Campo Grande 79070-900, Brazil; 4 Institute of Biosciences, Federal University of Mato Grosso do Sul, Campo Grande 79070-900, Brazil; 5 Department of Chemistry, Federal University of São Carlos, São Carlos 13565-905, Brazil

**Keywords:** photosensitizer, vector, photodynamic control, photodegradation, non-toxic

## Abstract

This study reports curcumin as an efficient photolarvicide against *Aedes aegypti* larvae under natural light illumination. Larval mortality and pupal formation were monitored daily for 21 days under simulated field conditions. In a sucrose-containing formulation, a lethal time 50 ($LT_{50}$) of 3 days was found using curcumin at 4.6 mg L^−1^. This formulation promoted no larval toxicity in the absence of illumination, and sucrose alone did not induce larval phototoxicity. The photodegradation byproducts (intermediates) of curcumin were determined and the photodegradation mechanisms proposed. Intermediates with *m/z* 194, 278, and 370 were found and characterized using LC-MS. The ecotoxicity of the byproducts on non-target organisms (*Daphnia*, fish, and green algae) indicates that the intermediates do not exhibit any destructive potential for aquatic organisms. The results of photodegradation and ecotoxicity suggest that curcumin is environmentally safe for non-target organisms and, therefore, can be considered for population control of *Ae*. *aegypti*.

## 1. Introduction

The *Aedes aegypti* mosquito is an insect vector responsible for transmitting different viruses that can cause, for instance, Chikungunya, Dengue, Yellow Fever, and Zika [1,2]. Insect vector population control has been tackled by mechanical, chemical, or biological methods [3,4,5]. Mechanical control is based, for example, on the elimination of reservoirs where vectors develop their life cycle. Chemical control is one of the most used strategies in larvae and adult mosquitoes due to its effectiveness and rapid action in arbovirus endemic or epidemic areas [3,6,7,8]. According to the World Health Organization (WHO), the insecticides used globally between 2010 and 2019 belong to various chemical classes to control the proliferation of the *Ae. aegypti* [7,9]. Larvicidal control can be performed using organophosphates, growth regulators, or biopesticides. Adulticides used for residual and space spraying belong to chemical classes such as carbamates, organophosphates, and pyrethroids [7,9,10]. Biological control could deal with insecticide-resistant insects, employing natural predators or pathogens to eradicate or decrease the vector population [3,11]. This type of control can be carried out using aquatic invertebrates (*Toxorhynchites* or copepods) or fish (*Gambusia* sp.) that feed on the larvae and pupae [11,12]. The bacterial pathogen *Bacillus thuringiensis israelensis* (Bti) has been recommended by the WHO as an important selective agent for the elimination of larvae [11]. These methods of controlling the *Ae aegypti* vector are strategies that are part of the integrated vector management (IVM) of increasing importance, which aims to reduce the emergence and transmission of new viruses [13,14].

Recent studies have shown photodynamic inactivation as a promising approach to controlling the *Ae. aegypti* larval population [15,16,17,18]. This photocatalytic method is based on the interaction between molecular oxygen (O_2_) and a photocatalyst agent, called a photosensitizer, activated by light, producing reactive oxygen species (ROS) that can kill target organisms by oxidizing them. Species such as hydrogen peroxide (H_2_O_2_), superoxide anions (O_2_**^.^**^−^), and hydroxyl radicals (·OH) may be produced by the type I photodynamic reaction (electron transfer mechanism) [19,20,21]. Singlet oxygen (^1^O_2_) production, which is highly reactive and cytotoxic, also occurs through the type II photodynamic reaction (energy transfer mechanism) [22].

Natural compounds isolated from plants have raised attention in searching for environmentally friendly photocatalysts. Curcumin extracted from *Curcuma longa* rhizomes is one of them, showing biological and photodynamic activity against microorganisms and insect vectors with ovicidal and larvicidal action [23]. Curcumin and its derivatives have shown larvicidal activity in vectors such as *Anopheles quadrimaculatus* (malaria vector) [23], *Culex quinquefasciatus* (bancroftian filariasis) [24], and *Cx. pipiens* (Japanese encephalitis) [25].

The photodynamic inactivation action of curcumin has been assessed against *Ae. aegypti* larvae [15,16]. High photolarvicidal and photo-ovicidal potential of curcumin were proved in either sucrose or D-mannitol. When associated with sucrose, photoactivated curcumin promoted larval mortality with a lethal concentration 50 after 24 h ($LC_{50-24h}$) in the 0.04–0.05 mg L^−^^1^ range. For D-mannitol formulations, a larval mortality was verified with $LC_{50-24h}$ values between 0.01 and 0.02 mg L^−1^ and a significant decrease in the hatching rate of eggs (10% at 100 mg L^−1^) [16]. Additionally, it has been reported that these formulations of curcumin with sugars (sucrose or D-mannitol) are non-toxic to the organisms *Daphnia magna* and *Danio rerio* [26].

Although studies have proved the photolarvicidal potential of curcumin to deal with *Ae. aegypti* larvae [16,26], points still need to be addressed before its application in the environment, including field tests and studies on the possible environmental impact related to its photodegradation. Curcumin is a well-established non-toxic molecule; however, there is a lack of information on the toxicity of photoproducts of curcumin. Although there are some works on the determination of curcumin derivatives [27,28,29], its photodegradation byproducts are still being unraveled [30], and the mechanism is still not clear [31]. Furthermore, most of the previous works evaluated a “non-pure curcumin”, which contains a mixture of three major curcuminoids (curcumin, demethoxy-curcumin, and bis-demethoxy-curcumin) obtained during the conventional extraction of curcumin [28]. Consequently, the photodegradation mechanism and byproducts from curcumin are affected by demethoxy-curcumin and bis-demethoxy-curcumin because of their different physicochemical and physiological properties [28].

This study synthesized pure curcumin and showed its efficient larvicidal photodynamic activity to control the *Ae. aegypti* population under field conditions (natural environment). Additionally, the present investigation discusses curcumin’s photodegradation mechanism, finding the byproducts (intermediates) and predicting their acute toxicity on non-target organisms (*Daphnia*, fish, and green algae).

## 2. Results and Discussion

### 2.1. Curcumin Localization in Aedes aegypti Tissues

**Figure 1:**
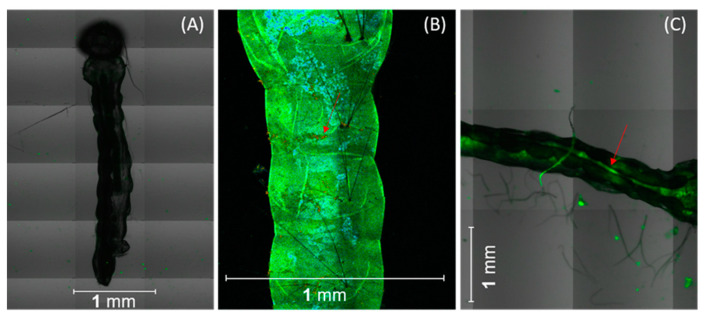
Representative confocal microscopy images of *Ae. aegypti* larvae: (**A**) non-subjected to synthetic curcumin with sucrose SCS (control) and exposed to SCS with curcumin, (**B**) attached to the larval body surface (arrow), and (**C**) located in the midgut. Scale bars represent 1 mm.

Confocal microscopy shows that the 10 larvae ingested synthetic curcumin formulation with sucrose (SCS), which led to accumulation in the abdominal segments, including the anterior, middle, and posterior regions (Figure 1). The results also revealed an adhesion of curcumin to the surface of the body of the larvae (Figure 1B). This accumulation may interfere with swimming performance, drastically reducing larval mobility [32]. Although curcumin was distributed throughout the entire gut, higher fluorescence intensity was observed in the midgut and the gastric cecum (Figure 1C). As recently reported by our research group, curcumin located in the larval midgut can cross the peritrophic membrane, which separates the food bolus from the midgut epithelium, causing irreversible damage to the intestinal epithelium [16].

### 2.2. Photodynamic Control

**Figure 2:**
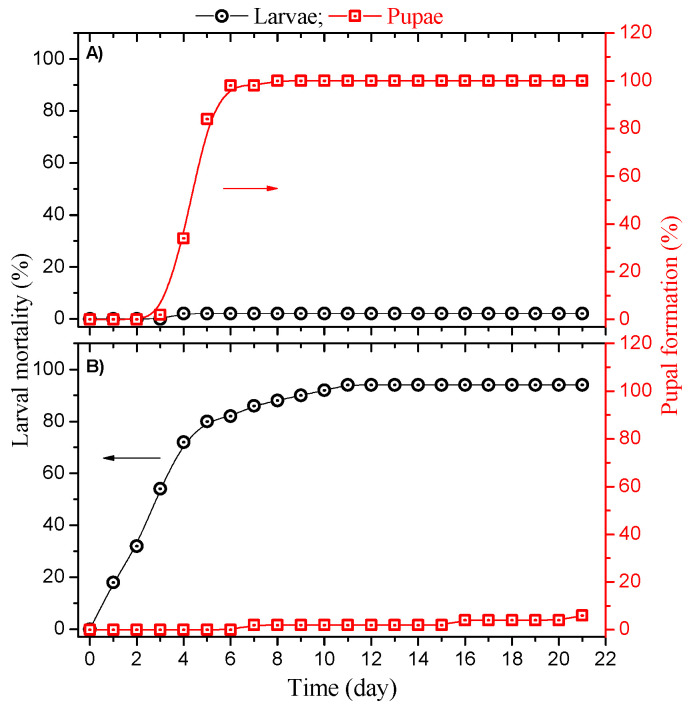
Pupal formation and larval mortality rate in the (**A**) control group submitted to sucrose formulation and (**B**) experimental group with curcumin/sucrose formulation as a function of the number of days in the field. Arrows indicate the *y*-axis for mortality (black) and pupal formation (red).

The pupal formation and mortality rate of the larvae added to the sucrose (control group) and SCS formulation (experimental group) as a function of the time are shown in Figure 2.

The L_3_ larvae in the control group reached the fourth instar (L_4_) between the third and fourth days in the field (data not shown). On the fifth day, 97% of the larvae evolved to pupal condition (Figure 2A). All larvae achieved the pupal stage on the seventh day. According to the literature, the larval life cycle of *Ae. aegypti* is about 7 days in a microbiota favorable for its development [33,34]. The pupation time occurred 13 days after the egg hatched in our experiments. The larvae took longer to reach the pupal stage, possibly because of the environmental conditions (Table S1, Supplementary Materials) [35]. Reinskind and Janairo reported that pupation could occur within 28 days because of a lack of nutrients and temperature conditions ranging from 26 (night) to 30 °C (day) [36]. Another study reported that temperature variations could influence larval development and pupation time [37].

Figure 2B shows the mortality and pupae formation in the experimental group with SCS formulation at a concentration of 4.6 mg L^−1^. Mortality increased over the days, reaching about 80% on the fifth day, and after 11 days, about 94% of the larvae died because of the photolarvicidal activity of curcumin under sunlight. The $LT_{50}$ at 3 days was calculated for the SCS formulation (4.6 mg L^−1^ of curcumin). Additionally, 6% of the larvae evolved to the pupal stage on the twenty-first day, so curcumin also impacted the pupation of the larvae that remained alive, considering that all L_3_ larvae in the control group evolved to the pupal stage from the seventh day of monitoring. Hence, curcumin delayed pupation even when it did not induce larval death. Mezzacappo et al. also found that curcumin affects the development of *Ae. aegypti* from larvae to adults, postponing the start of the pupation phase [38]. SCS formulation and sucrose did not show significant toxicity in the dark, as determined in the laboratory trial. Moreover, no significant larval mortality in unlighted groups containing curcumin and sucrose has been reported (data not shown) [16].

The larvicidal activity of curcuminoids has been investigated in various insect vectors. Curcumin, in its natural form, exhibited a $LC_{50}$ of 19.07 and 32.5 mg L^−1^ in *Cx. pipiens* and *An. quadrimaculatus* larvae, respectively [23,25]. Furthermore, the researchers informed the $LC_{50-24h}$ of natural curcumin (49.3 mg L^−1^) and its essential oil (115.6 mg L^−1^) for the *Ae. aegypti* vector [23,39].

The phototoxic effects of curcumin on *Ae. aegypti* larvae were demonstrated in laboratory tests using natural turmeric (NT), synthetic curcumin (SC), and SCS with concentrations between 5 and 25 mg L^−1^ and solar irradiance from 30 to 60 mW cm^−2^. NT, SC, and SCS curcuminoids showed high photolarvicidal activity with $LC_{50-3h}$ of 20.0, 11.6, and 2.2 mg L^−1^, respectively. In the absence of irradiation, no larval mortality was observed at concentrations below 25 mg L^−1^ [15].

In another study, Souza et al. investigated the photolarvicidal capacity of the SCS formulation (0.005–0.45 mg L^−1^) on *Ae. aegypti* larvae directly irradiated [16]. After 8 h of exposure to SCS and solar radiation, the container was kept in the dark for 16 h to assess the larvae mortality with 24 h. A photolarvicidal effect with an $LC_{50-24h}$ of 0.04 mg L^−1^ was found. In the absence of radiation, the nonilluminated group did not show significant larval mortality. It is relevant to point out that the experimental conditions adopted for that laboratory study differed from those employed in this investigation, such as exposure time to sunlight, the SCS concentration, container, and environment. Here, the photolarvicidal potential of SCS for *Ae. aegypti* was demonstrated in its habitat under natural conditions with a lethal time ($LT_{50}$) of 3 days using 4.6 mg L^−1^ of SC in the SCS formulation.

### 2.3. Curcumin Photodegradation Byproducts

The chromatogram peaks corresponding to the protonated molecules [M+H]+ allowed the investigation of the intermediates collected from the samples at 0, 90, and 180 min of photodynamic treatment, produced by direct photolysis of the SC (Figure S1, Supplementary Materials). Thus, as a reference, the photodegradation byproducts were evaluated using the sample at the beginning of the treatment (0 min). Table 1 summarizes the proposed molecular structures and formulas related to the mass/charge (*m/z*) ratio of the protonated molecules and the respective retention times. In the photodegradation experiments, SC was diluted in an aqueous solution as well as anhydrous ethanol to evaluate the effect of hydroxyl on the curcumin photodegradation mechanism. The mass spectra exhibited the same *m/z* peaks regardless of the medium; hence, the water medium does not contribute significantly to the photodegradation.

In photodegradation processes, hydroxylations of the organic compound can be promoted by the superoxide (O_2_**^.^**^−^) radicals (Equations (1) and (2)) [40].

R+ hν → R*(1)

R* + O_2_ → R ^+^ + O_2_**^.^**^−^ (2)

**Figure 3:**
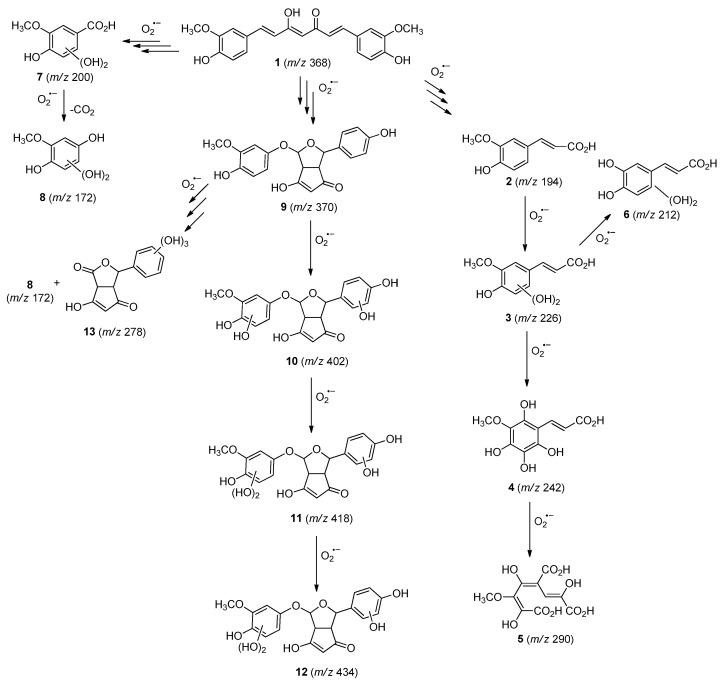
Proposal for formation of curcumin photodegradation byproducts.

The hydroxylation of arylated organic compounds caused by O_2_**^.^**^−^ radicals contributes to the formation of phenols, alcohols, aldehydes, and carboxylic acids [41,42,43,44]. Twelve intermediates with *m/z* 172, 194, 200, 212, 226, 242, 278, 290, 370, 402, 418, and 434 during the curcumin photodegradation were identified. Figure 3 displays the proposed photodegradation byproducts based on these intermediates.

From the curcumin **1** *m/z* 368, the intermediate **2** *m/z* 194 (ferulic acid) was obtained due to the action of the O_2_**^.^**^−^ radical [45]. Next, attacks by O_2_**^.^**^−^ led to highly hydroxylated byproducts with *m/z* 172, 200, 212, 226, and 242, such as di- and trihydroxylated aromatic compounds [42,46,47]. Photodegradation of **2** yielded **3** *m/z* 226, followed by **4** *m/z* 242, a trihydroxylated compound. C–C cleavage of the aromatic ring of **4** by the radicals O_2_**^.^**^−^ and hydroperoxyl (HO_2_·) produced the byproduct **5** *m/z* 290, raising the carboxylic acid functional group [42,47,48,49].

The formation of **6** with *m/z* 212 could be attributed to the C–O bond cleavage of the methoxy group linked to the aromatic ring because of the reaction between O_2_**^.^**^−^ and the byproduct **3** (*m/z* 226). O_2_**^.^**^−^ radicals could also lead to the intermediate **7** (*m/z* 200) with a lower carbon chain. By losing carbon, this byproduct **7** allowed the formation of **8** with the lowest molecular weight (*m/z* 172).

During photodegradation, O_2_**^.^**^−^ also could capture hydrogen, giving rise to **9** *m/z* 370 (demethoxybicyclopentadione) [29]. Subsequently, O_2_**^.^**^−^ hydroxylates the aromatic rings, forming di- and monohydroxylates **10**, **11**, and **12** with *m/z* 402, 218, and 434, respectively. The reaction of the O_2_**^.^**^−^ with **9** (*m/z* 370) enabled the C–O bond cleavage of the aromatic ether group and the appearance of the byproducts **13** (*m/z* 278) and **8** (*m/z* 172). Therefore, the stages of the photodegradation process of curcumin are based on the O_2_**^.^**^−^ generation that promotes the formation of highly hydroxylated compounds [42,50,51].

### 2.4. Prediction of Ecotoxicity of Intermediates from Curcumin Photodegradation

As hydroxylated intermediates present low acute toxicity (Table 2) due to the reduced tendency to be liposoluble, only three leading intermediates, **2**, **13**, and **9** (*m/z* 194, 278, and 370, respectively), were selected for the ecotoxicity assessment [42,50,51]. The partition coefficients ($log\;K_{O/w}$) are also shown, which were used to evaluate the solubility of compounds in water, their tendency to interact with structures, and their permeability in cell membranes [51,52].

Compounds with $log\;K_{O/w}$ greater than 3 are considered highly liposoluble and, therefore, reveal a greater tendency to be adsorbed in the organic phase of living organisms [42,52]. The values are less than 3 for the intermediates evaluated, suggesting their low liposolubility and affinity for living organisms and, thus, their permanence in water until photodegradation.

The high $LC_{50}$ and $EC_{50}$ associated with the hydroxylation of these intermediates resulted in TU less than 0.4—between 5.8 × 10^−3^ and 4.3 × 10^−5^; hence, these compounds are non-toxic to *Daphnia*, fish, and green algae [53].

Curcumin and its derivatives have been widely used in the food, pharmacological, and cosmetic industries [54]. Clinical studies have shown that curcumin is safe for humans even when a 12 g dose is administered orally every day for 3 months [55,56,57]. The results reported here show that the byproducts obtained in the photobleaching of curcumin do not have any toxic potential for the aquatic organisms *Daphnia*, fish, and green algae.

## 3. Materials and Methods

### 3.1. Photosensitizer

Curcumin (≥98%) was synthesized using continuous flow technologies at the Federal University of São Carlos. The organic synthesis team developed this assisted machine protocol from the Center of Optics and Photonics (CePOF) [58]. Sucrose (≥99%, Sigma-Aldrich, St. Louis, MO, USA) was mixed with curcumin to increase water solubility and palatability, acting as a phagostimulant [16,59,60]. The mixture was formulated in the proportion of 1% of SC and 99% of sucrose (*m*/*m*) [16]. The formulation of SCS was solubilized in an aqueous medium at a concentration of curcumin and sucrose of 4.6 and 445.4 mg L^−1^, respectively (curcumin-containing group). A control group was also tested, which contained only sucrose at 445.4 mg L^−1^ in an aqueous solution.

### 3.2. Photodynamic Control Bioassays

Larvae were obtained from eggs of *Ae. aegypti* (Rockefeller lineage). Bioassays followed the recommendations of the WHO with modifications [61]. Eggs were deposited on filter paper and placed on plastic trays with approximately 2 L of tap water until hatching. After, the larvae were fed with macerated fish feed (Alcon^®^, Santa Catarina,, Brazil) dissolved in water. The trays were kept at 25 °C in a biological oxygen demand oven with humidity (60 ± 5)% and a photoperiod of 10:14 h dark:light. Third instar larvae (L_3_) were used to evaluate the photoinactivation potential of SCS in field experiments.

The localization of curcumin in the *Ae. aegypti* was assessed using a Zeiss inverted fluorescence confocal microscope (LSM780, Jena, Germany) in the so-called channel mode with a 20× objective. The fluorescence images were obtained by collecting emission from curcumin in the 520–550 nm range under 405-nm excitation. Then the L_3_ *Ae*. *aegypti* larvae were added to a solution with 25 mg L^−1^ of curcumin. After 12 h in the dark, the larvae were washed three times in distilled water, transferred to microscopic slides, and covered with coverslips. The experiment was conducted in triplicate, and about 10 larvae were examined per experiment.

**Figure 4:**
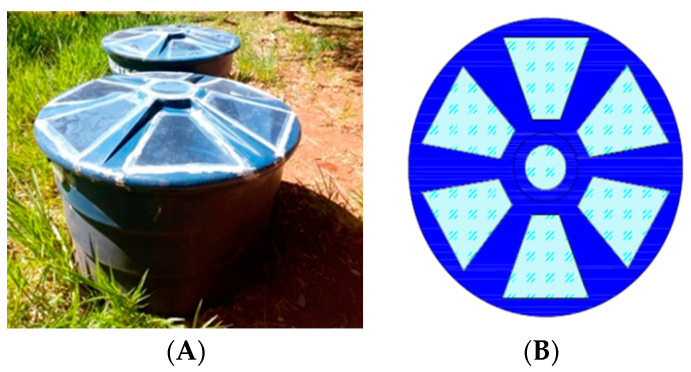
(**A**) Water tanks; (**B**) tank cover illustration.

The field experiments of photodynamic inactivation using solar radiation were performed on the meteorological station at a 534 m altitude, latitude 20°50′51′′ W, and longitude 54°61′73′′ NW (Campo Grande, MS, Brazil) following Vilarinhos et al. 2003 with modifications [61,62]. Two 500 L water tanks were adapted to store the control and experimental groups (Figure 4). The covers were cut, and the trapezoidal openings were covered with transparent polyethylene acrylic to allow sunlight into the tanks (Figure 4). Although each container had a capacity of 500 L, only 250 L solutions were used, which resulted in a depth of water of 38 cm.

The sucrose and SCS (4.6 mg L^−1^ curcumin) formulation were diluted directly in 250 L of tap water in the tanks of the control and experimental groups, respectively. The concentration of 4.6 mg L^−1^ was adopted based on preliminary tests in the laboratory employing five concentrations (1.8; 4.6; 5.4; 7.2; 14.4 mg L^−1^) under 450 nm illumination. All tested concentrations induced photolarvicidal effect; nevertheless, an intermediate concentration was preferred based on the preliminary results. The efficiency of photodynamic inactivation of *Ae*. *aegypti* larvae using SCS formulation and solar radiation was evaluated in a simulated habitat. On reaching the third instar (L_3_, 6 days after hatching), a total of 50 larvae of *Ae*. *aegypti* were placed in water tanks to start the monitoring. The larvae were fed with 2.5 mg of macerated fish feed Alcon^®^ on alternate days according to the number of larvae in each tank. The literature indicates 10 mg L^−1^ of food at intervals of up to 2 days for 25–100 larvae [61].

Larval mortality and pupal formation were checked daily at 10 AM for 21 days during the summer. The solutions were slightly agitated; larvae that emerged motionless were considered dead and collected by a Pasteur pipette. Dead larvae and pupae formed were counted and removed. During larval mortality monitoring, the luminous intensity reaching the water tank was also measured daily. The luminosity at 10 AM varied from 240 to 136,800 lx during the 21-day experiments (Table S1, Supplementary Materials). The water temperature was monitored in the tank, varying between (25.0 ± 0.5) and (29.0 ± 0.5) °C throughout the experiment, while the ambient temperature ranged from (24.0 ± 0.5) to (33.0 ± 0.5) °C (Table S1, Supplementary Materials).

The average larval mortality data were submitted to probit analysis to obtain the lethal to one-half time ($LT_{50}$) with a corresponding 95% confidence interval using Origin v.2022 software.

### 3.3. Photodegradation

The study of photodegradation byproducts was carried out based on two solutions containing SC diluted in distilled water and anhydrous ethanol. Both solutions were obtained by solubilizing curcumin in ethanol (HPLC 99.9%, Sigma Aldrich, St. Louis, MI, USA) at a concentration of 900 µg mL^−1^. Distilled water (100 mL) and 1.6 mL of the stock solution resulted in an aqueous solution of curcumin with a concentration of 14.4 µg mL^−1^. Another solution was prepared from 1.6 mL of the stock solution by adding ethanol to obtain a solution with the same concentration of curcumin.

Photodegradation experiments were performed in a solar box (Abet Technology 10500) with a Xenon lamp (300 W) and an AM1.5G filter. A 45 mL solution of curcumin (14.4 μg mL^−1^) was subjected to an irradiance of 100 mW cm^−2^ (132,000 lx) for 3 h. Aliquots were taken at 0, 90, and 180 min to monitor the photodegradation byproducts using liquid chromatography with a mass spectrometer (LC-MS) [63,64].

### 3.4. Liquid Chromatography with Mass Spectrometer (LC-MS)

The solutions, prepared in the proportion of 5 mL of sample to 5 mL of methanol (CL-MS grade, Panreac), were injected (1 µL) in a liquid chromatograph/mass spectrometer (UFLC Shimadzu LC-20AD—IES Detector-Q-QTOF microTOFIII (Bruker Daltonics)) equipped with a Phenomenex onyx monolithic C-18 column (100 × 3.0 mm). The operating parameters were set up following the method reported by Da Silva et al., 2018, and Da Rosa et al., 2019 [65,66]. The system operated in positive electrospray ionization mode with a spray voltage of 0.8–1.2 V and a capillary voltage of 3500 V (200 °C). Mass spectra were obtained within the *m/z* range 120–1300. Gradient elution with water (phase A) and acetonitrile (phase B), both with 0.1% formic acid, was performed with a flow rate of 0.3 mL min^−1^. The gradient ramps were: 0–2 min, 0% to 3% B; 2–25 min, 3% to 25% B; 25–40 min from 25% to 80% B; and 40–48 min, from 80% to 3% B.

### 3.5. Prediction of Acute Toxicity of Intermediates for Aquatic Organisms

Acute toxicity unit (TU), lethal concentration 50 ($LC_{50}$), and half-maximal effective concentration ($EC_{50}$) of the primary hydroxylated intermediates (*m/z* 194, 278, and 370) formed by the photodegradation of curcumin on the non-target organisms were estimated using the ecological structure-activity relationship (ECOSAR) model (version 1.11, Washington, DC, USA) [67]. The $LC_{50}$ concentration values for *Daphnia* and fish were determined as well as the concentration ($EC_{50}$) that inhibits the growth of the green algae population by 50%. Consequently, it is possible to estimate the acute toxic unit (TU), defined as $\left(1/LC_{50}\right)\times 100$ or $\left(1/EC_{50}\right)\times 100$ [68].

ECOSAR was developed by the Office of Pollution Prevention and Toxics, U.S. Environmental Protection Agency (U.S. EPA/OPPT), and collaborators, to predict the aquatic toxicity of new chemicals for industrial applications, pointing to those requiring further toxic testing and characterization [68,69]. The model takes into account the linear relationship between the calculated $\log K_{\mathrm{OW}}$ values and the related logarithm of the measured toxicity values for a given training set of compounds within each class of interest. For freshwater fish data, species such as bluegill sunfish (*Lepomis macrochirus*) and fathead minnow (*Pimephales promelas*) are included; for freshwater invertebrates, species involve *Daphnia magna* or *Daphnia pulex*; and for algae, *Desmodesmus subspicatus* or *Pseudokirchneriella subcapitata* [68,69]. Therefore, it is important to highlight that the terms fish, *Daphnia*, and green algae represent a set of species adopted in the training set of the program to assess the toxicity values of chemicals on the general trophic levels that they embody.

## 4. Conclusions

Curcumin was formulated with sucrose and showed significant photoactivity against larvae of *Ae. aegypti* under field conditions. This study confirmed the potential of this natural-based photosensitizer to mitigate mosquito growth in field conditions. It also identified byproducts from photodegradation in a natural environment with the proposal of degradation byproducts. Based on LC-MS studies, these compounds were suggested and revealed the main ones with *m/z* 194, 278, and 370. Ecotoxicity predictions for these intermediates do not indicate any harmful potential for species *Daphnia*, fish, and green algae. Therefore, curcumin-promoted oxidative storms (photodynamic effect) under a broad light spectrum (sunlight) can simultaneously induce the death of *Ae. aegypti* larvae and a fast and efficient photodegradation of curcumin, yielding water-soluble byproducts that are not potentially ecotoxic. Consequently, the present work proved that curcumin could be used as an environmentally safe photosensitizer to deal with the larval population of *Ae. aegypti* under field conditions.

## Figures and Tables

**Table 1:** Proposed structures of the produced byproducts obtained from direct photolysis of curcumin, molecular formulas with the respective *m/z*, and retention time.

Compound	Accurate Mass [M+H]+	Retention Time (min)	Molecular Formula	Proposed Structure
172 *m/z*	173.0103	1	C_7_H_8_O_5_	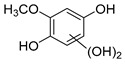
194 *m/z*	194.9671	1.1	C_10_H_10_O_4_	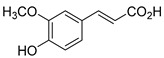
200 *m/z*	201.0466	1	C_8_H_8_O_6_	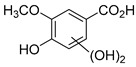
212 *m/z*	212.9753	1.1	C_10_H_8_O_6_	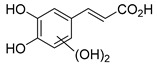
226 *m/z*	226.9489	1	C_10_H_10_O_6_	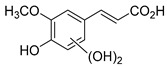
242 *m/z*	242.9826	1.1	C_10_H_10_O_7_	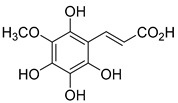
278 *m/z*	279.0721	1	C_13_H_10_O_7_	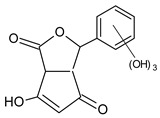
290 *m/z*	290.9712	1	C_10_H_10_O_10_	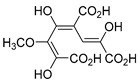
370 *m/z*	370.9319	1.1	C_20_H_18_O_7_	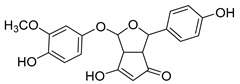
402 *m/z*	402.8989	1.1	C_20_H_18_O_9_	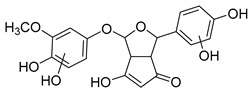
418 *m/z*	418.8622	1.1	C_20_H_18_O_10_	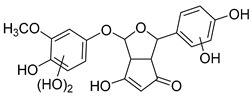
434 *m/z*	434.8924	1.1	C_20_H_18_O_11_	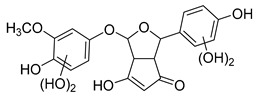

**Table 2:** Predicted acute toxicity to *Daphnia*, fish, and green algae for some intermediates of curcumin photodegradation: $LC_{50}$, $EC_{50}$, $log\;K_{O/w}$, water-solubility, acute toxic unit (TU), and toxicity classification.

	Compound	2 (194 *m/z*)	13 (278 *m/z*)	9 (370 *m/z*)
Organism	Structure	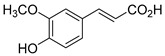	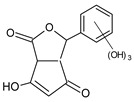	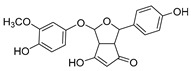
*Daphnia*	$LC_{50}$ (mg L^−1^)	287.5	2.3 × 10^4^	988.16
	TU	3.5 × 10^−3^	4.3 × 10^−5^	1.2 × 10^−3^
Fish	$LC_{50}$ (mg L^−1^)	534.4	7.3 × 10^3^	1.9 × 10^3^
	TU	1.9 × 10^−3^	1.4 × 10^−4^	5.3 × 10^−4^
Green algae	$EC_{50}$ (mg L^−1^)	171.3	1.8 × 10^4^	525.5
	TU	5.8 × 10^−3^	5.4 × 10^−5^	1.9 × 10^−3^
	Toxicity	Non-toxic	Non-toxic	Non-toxic
	$log\;K_{O/w}\;$	1.42	−1.93	1.12

## Data Availability

Not applicable.

## Supplementary Materials

The following supporting information can be downloaded at: https://www.mdpi.com/article/10.3390/molecules27175699/s1, Figure S1: Mass spectra of intermediates from curcumin photodegradation obtained in ethanol at 0, 90, and 180 min; Table S1: Temperature and irradiance measured daily at 10 A.M. at the experimental site.
