# Supplementary material for: Environmentally Safe Photodynamic Control of Aedes aegypti Using Sunlight-Activated Synthetic Curcumin: Photodegradation, Aquatic Ecotoxicity, and Field Trial
Source: Molecules. 2022 Sep 4;27(17):5699. doi: 10.3390/molecules27175699 (PMC9457702; doi:10.3390/molecules27175699)
Supplement: Supplementary file 1 [file molecules-27-05699-s001.zip › molecules-1837555-supplementary.pdf]

Supplementary Information

Environmentally Safe Photodynamic Control of *Aedes aegypti* Using Sunlight-Activated Synthetic Curcumin: Photodegradation, Aquatic Ecotoxicity, and Field Trial

Table S1. Temperature and irradiance daily measured at 10 AM in the experimental site.

Experimental conditions			
**Day**	**Environmental Temperature (°C)**	**Water Temperature (°C)**	**Irradiance (Lux)**
1	28	27	240
2	30	28	1,128
3	29	27	7,410
4	24	25	11,240
5	28	25	112,400
6	25	26.8	13,600
7	29	26	9,190
8	28	27	12,370
9	26	28	4,600
10	31	27	12,270
11	27	28	2,770
12	30	27	11,200
13	30	28	22,900
14	33	29	114,400
15	29	28	6,290
16	30	29	136,800
17	28	27	6,800
18	28	26	6,310
19	28.5	26	22,100
20	28	25	12,910
21	28	26	21,000

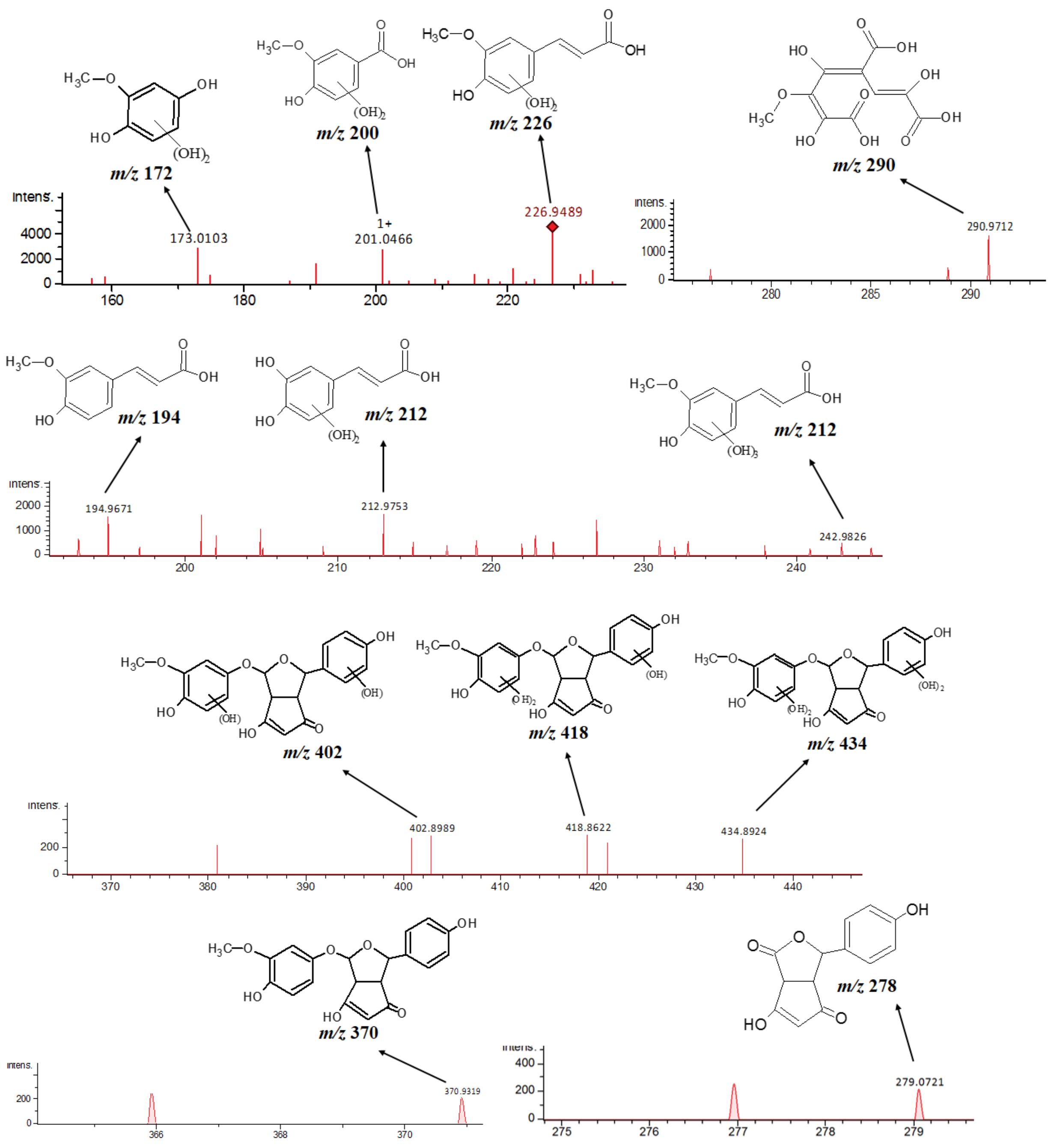

Figure S1. Mass spectra of intermediates from curcumin photodegradation obtained in ethanol at 0, 90, and 180 min.
